# Vasculitis

**DOI:** 10.1186/1546-0096-12-S1-I20

**Published:** 2014-09-17

**Authors:** Pavla Dolezalova

**Affiliations:** 1 Paediatric Rheumatology Unit, Department of Paediatrics and Adolescent Medicine, General University Hospital and 1st Faculty of Medicine, Charles University in Prague , Praha, Czech Republic

## 

Primary systemic vasculitides (PSV) in children belong to the most challenging conditions in paediatric rheumatology practice. The spectrum of vasculitis affecting children as well as their clinical presentations differ from that of adults. Apart from relatively common Henoch-Shönlein purpura and Kawasaki disease, chronic PSV are extremely rare in childhood. They include mainly childhood polyarteritis nodosa (cPAN), Takayasu arteritis (cTA) and granulomatosis with polyangiitis (cGPA, formerly Wegener´s granulomatosis). From organ-specific diseases, primary CNS angiitis (PACNS) has been increasingly recognised in children over the recent years. Differential diagnosis of vasculitis covers wide spectrum of systemic diseases of inflammatory and neoplastic origin as well as etiologically heterogeneous group of non-inflammatory conditions reffered to as “pseudovasculitis”. In general, diagnostic confirmation of vasculitis requires histopathological or angiographic evidence of vascular involvement. Main diagnostic and treatment principles and classification criteria for the main vasculitides will be reviewed.

Principles of vasculitis assessment have been formulated by the OMERACT (Outcome Measures in Rheumatology) Vasculitis Working Group.^1^ Reversible features of acute morbidity directly related to the underlying inflammation are captured by the disease activity domain while irreversible consequences of previous active disease or long-term sequleae of treatment adverse effects form the basis of the disease damage domain. Physical function in terms of the degree of disability as well as psychosocial functioning including educational and vocational aspects are additional important components of patient-reported outcomes covered by the domain of health-related quality of life (HRQL). Paediatric-specific tools for vasculitis disease activity and damage assessment have been derived from adult instruments. Principles of Paediatric Vasculitis Activity Score (PVAS) and Paediatric Vasculitis Damage Index (PVDI) will be explained^2,3^.

Availability of childhood vasculitis classification and disease assessment tools has enabled initiation of the first paediatric vasculitis clinical trials. Ongoing international activities in the field of vasculitis include prospective disease registries that would allow update of disease classification and development of diagnostic criteria as well as improvement and validation of disease assessment tools.

## Disclosure of interest

P Dolezalova Grant / Research Support from: Novartis, Roche, Abbvie, Pfizer, Consultant for: Roche.

